# Morning physical activity may be more beneficial for blood lipids than afternoon physical activity in older adults: a cross-sectional study

**DOI:** 10.1007/s00421-024-05526-y

**Published:** 2024-06-14

**Authors:** Hyeon-Ki Kim, Yuga Kimura, Masaki Takahashi, Takashi Nakaoka, Yosuke Yamada, Rei Ono, Shigenobu Shibata

**Affiliations:** 1 National Institute of Biomedical Innovation, Health and Nutrition, 3-17 Senriokashimmachi, Settsu-shi, Osaka 566-0002 Japan; 2 School of Advance Science and Engineering, Waseda University, Tokyo, Japan; 3 Institute for Liberal Arts, Tokyo Institute of Technology, Tokyo, Japan; 4 Japan Organization of Occupational Health and Safety, Kanagawa, Japan; 5 Faculty of Science and Engineering, Waseda University, Tokyo, Japan; 6 Graduate School of Biomedical and Health Sciences, Hiroshima University, Hiroshima, Japan

**Keywords:** Physical activity pattern, Number of steps, Moderate-vigorous physical activity, Cardiovascular risk factors, Blood lipids

## Abstract

**Background:**

The effect of differences in daily physical activity patterns on blood lipids has not been determined. This study examines the effects of the differences in free-living daily physical activity patterns (amount and intensity) on blood lipid levels in older adults.

**Methods:**

This cross-sectional study included 51 older participants (71.8 ± 0.6 years, men = 8, women = 43). A triaxial accelerometer was used to assess physical activity patterns. The time from awakening to bedtime for each participant was used for group classification based on the amount (number of steps) and intensity (moderate-to-vigorous physical activity, MVPA) of physical activity. The morning step group (M Step) was defined as those who took more steps in the morning, and the afternoon step group (A Step) was defined as those who took more steps in the afternoon. The same method was used for MVPA (morning MVPA: M MVPA; afternoon MVPA: A MVPA). Blood samples were collected at the start of the study to determine blood lipid levels.

**Results:**

Number of steps taken showed a trend toward lower low-density lipoprotein cholesterol (LDL-C) levels in the M Step group compared with the A Step group. The LDL/high-density lipoprotein (HDL) ratio was significantly lower in the M Step group than the A Step group (*p* < 0.05). The M MVPA group also had higher HDL-C levels and significantly lower LDL/HDL ratios than the A MVPA group *(p* < 0.05).

**Conclusions:**

These results suggest that compared with afternoon physical activity, daily morning physical activity (amount and intensity) is more effective in improving blood lipid levels.

**Supplementary Information:**

The online version contains supplementary material available at 10.1007/s00421-024-05526-y.

## Introduction

Human life expectancy is rapidly increasing, and the number of older people is projected to double by 2050 (Nations 2017). Aging increases the risk of cardiovascular diseases (CVDs), a major cause of increased mortality (Chodzko-Zajko et al. 2009). Furthermore, physical inactivity is more prevalent among older adults than younger adults, and a significant proportion also experience obesity (Fakhouri et al. 2012). Therefore, age-related physical inactivity increases the risk of CVDs (Chodzko-Zajko et al. 2009). Conversely, regular physical activity is effective in improving cardiovascular disease-related factors, such as blood lipids and blood pressure (Chodzko-Zajko et al. 2009). The results of the epidemiological studies and meta-analyses on the association between increased physical activity and CVD demonstrated the benefits of physical activity on CVD and mortality (Nelson et al. 2007). A previous study also showed that the biological effects of physical activity levels varied, with lower blood lipid levels and blood pressure reported in the more physically active group than the less physically active group (Muga et al. 2019). Considering the biological effects of physical activity, increased physical activity could improve blood lipid levels and blood pressure.

Regular physical activity is important for maintaining good health. Therefore, moderate-intensity physical activity for at least 150 min/week is recommended (Bull et al. 2020; Donnelly et al. 2009; Li et al. 2014; Piercy et al. 2018). Recent research provides insight into maximizing health benefits through various aspects of physical activity, including intensity and type (Lear et al. 2017; Millard et al. 2021; Strain et al. 2020). Additionally, the effects of meal and sleep timing on health are being elucidated (Acosta-Rodríguez et al. 2021; Fan et al. 2020). However, research on the health effects of timing physical activity is scant. Moreover, previous studies showed that the time distribution of moderate-to-vigorous physical activity (MVPA) accumulated during the day (physical activity patterns) was an important health determinant (Qian et al. 2021; Xu et al. 2019). Previous studies on mice revealed that the metabolic response to exercise varied with the time of day, which suggested a time-dependent beneficial effect of exercise via the endogenous circadian system (Ezagouri et al. 2019; Sato et al. 2019). Several studies on humans found that exercise timing regulated weight loss (Willis et al. 2020), lipolysis (Kim et al. 2015), glucose and blood lipid control (Kim et al. 2022; Savikj et al. 2019), and acute cardiovascular response (Scheer et al. 2010). However, while animal studies have yielded consistent results regarding the effects of exercise timing on metabolism (Ezagouri et al. 2019; Sato et al. 2019, 2022), human studies have yielded conflicting results (DiPietro et al. 2013; Kim et al. 2022; Moholdt et al. 2021; Savikj et al. 2019). For example, a previous study showed that the metabolic effects of exercise are more robust in the morning than at night (Ezagouri et al. 2019). A previous human study also showed that compared with afternoon exercise, morning exercise was more effective in controlling blood glucose levels (Gomez et al. 2015). However, another previous study reported a greater decrease in blood glucose levels with afternoon exercise compared with morning exercise (Kim et al. 2022; Savikj et al. 2019). Therefore, the optimal time of day for physical activity for human health remains unknown. Additionally, these studies assessed the effects of structured exercise periods. Free-living physical activities can include household chores, commuting, or work-related activities. Therefore, clarifying the effects of the temporal distribution of physical activity that accumulates throughout the day is important.

Accelerometers provide an objective and continuous assessment of physical activity in free-living environments (Atienza et al. 2011). Most studies on physical activity that used accelerometers averaged the measures over the entire wear time (e.g., average daily physical activity (number of steps) and duration of activity intensity) (Manns et al. 2015). However, accelerometer data are recorded at a considerably higher resolution (e.g., more than once per minute), making it important to examine physical activity patterns more comprehensively (Schrack et al. 2014; Xiao et al. 2015). Many previous studies on physical activity patterns and health-related outcomes focus on disease, while few focus on disease-related blood indices. Therefore, several issues regarding this topic remain unclear (Cooper et al. 2017; Huisingh-Scheetz et al. 2018; Varma and Watts 2017). Additionally, physical activity patterns in daily life differed at different times based on individual lifestyles and social environments (Evenson et al. 2012; Sallis et al. 2006). Therefore, the heterogeneity of physical activity required further investigation.

Hence, this study examines the effects of different physical activity patterns (amount and intensity) on blood lipid levels in older adults aged 65 years and above.

## Methods

### Participants

The participants included 51 older people (65–82 years old, 8 men and 43 women) who lived in the Tokyo and Saitama prefectures. Participants do not smoke, do not exercise regularly, and have no medical conditions confirmed by medical examination. Exercise habits were defined as continuous physical activity, at least three times per week, for at least 30 min per session (Butcher et al. 2008). The exclusion criteria included those who were diagnosed with diabetes and dyslipidemia by physical examination, took medication, or already active in another research intervention program. However, none met the exclusion criteria. Hence, all the participants were included. Additionally, none had difficulties with mobility or activities of daily living. Before the study, all the participants were briefed about the study and its safety, and informed written consent was obtained. The study was conducted in accordance with the tenets of the Declaration of Helsinki and its future amendments. This study was approved by the Waseda University’s Ethics Committee (approval no. 2019-194).

### Study design

**Fig. 1:**
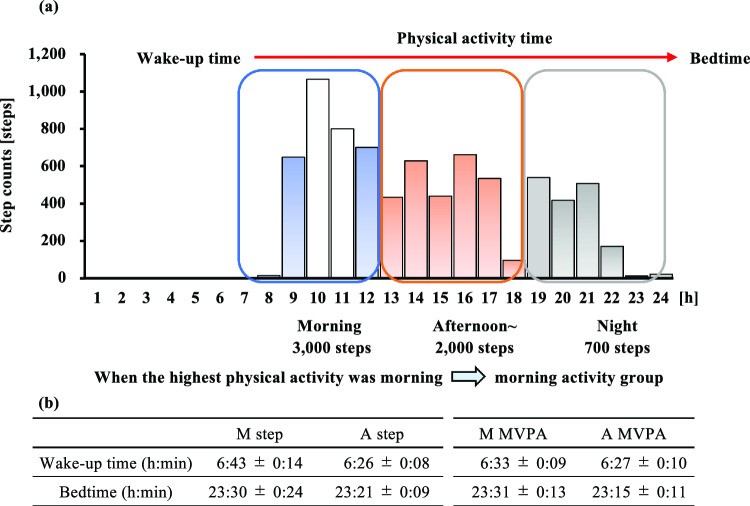
Example of grouping by physical activity pattern (**a**) and wake-up time and bedtime (**b**). MVPA: moderate-to-vigorous physical activity, M step: morning step group, A step: afternoon step group, M MVPA: morning MVPA group, A MVPA: afternoon MVPA group

**Fig. 2:**
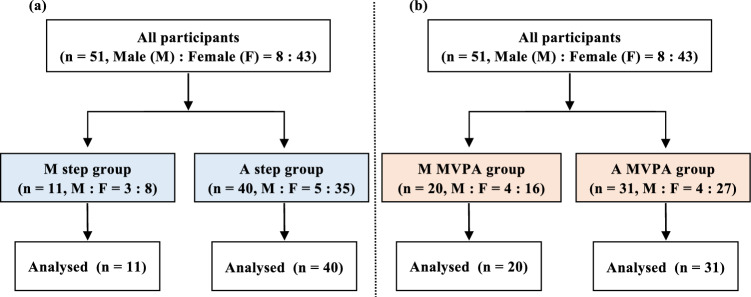
Flow diagram of the participants. Grouping using the number of steps (**a**) and MVPA (**b**). MVPA: moderate-to-vigorous physical activity, M step: morning step group, A step: afternoon step group, M MVPA: morning MVPA group, A MVPA: afternoon MVPA group

This was a cross-sectional study. A triaxial accelerometer was used to examine the relationship between physical activity patterns and blood lipid levels. Grouping was performed by calculating each participant's physical activity time from awakening to bedtime. The activity time was then divided into the third quantile (Fig. 1a). Wake and bedtimes for each group are shown in Fig. 1b. No significant differences were found between the morning and afternoon groups in terms of wake and bedtimes. The groups were also divided according to the amount (number of steps) and intensity (MVPA) of physical activity (Fig. 2a and b, respectively). The reason for grouping by amount or intensity was that MVPA was not necessarily higher in the morning, although the amount of accumulated physical activity (number of steps) was higher in the morning. The morning step group (M step, n = 11) was defined as those who took more steps in the morning, and the afternoon step group (A step, n = 40) was defined as those who took more steps in the afternoon. The same method was used for MVPA (morning MVPA: M MVPA, n = 20; afternoon MVPA: A MVPA, n = 31). In addition, if the number of physical activity steps or MVPA accumulation was greater at night, the participant was placed in a night steps (N steps)/night MVPA (N MVPA) group. However, there were no participants in the night group. Therefore, the morning and afternoon groups were compared.

### Physical activity levels

All participants were asked to wear a triaxial accelerometer (Active-style Pro HJA-750C; Omron Healthcare Co., Ltd., Kyoto, Japan) on their waist for one week. Accelerometers were worn every day, from morning to night, except during showering and bedtime. The device determined the level of intensity (METs, metabolic equivalents) generated by activity every 10 s from 0–8 METs (where 0 was the lowest and 8 was the highest). Data from the participants, who wore the accelerometer for at least 10 h (600 min) daily for at least four weekdays and a day on the weekend, were included. Apart from that, the duration of daily moderate to vigorous physical activity (MVPA) was calculated and used to estimate weekly activity by calculating a weighted average of daily weekday and weekend activities. We used MVPA and number of steps required for the evaluation. All minute records of ≥ 3 METs were classified as MVPA (Kim et al. 2022).

### Anthropometry

Body mass and height were measured to the nearest 0.1 kg and 0.1 cm via a digital scale (Inbody 230, Inbody Inc., Tokyo, Japan) and wall-mounted stadiometer (YS-OA, As One Corp., Osaka, Japan), respectively. BMI was calculated as weight (kg) divided by height squared (m^2^).

### Chronotype

Chronotype (morningness to eveningness) was evaluated using the morningness–eveningness questionnaire (MEQ) (Horne and Ostberg 1976). The MEQ is composed of 19 items related to sleep habits, sleepiness, and the preferred time for daily performance. Scores ranged from 16–86 points. Participants were divided into the following three chronotype groups: morningness (59–86), intermediate (42–58), and eveningness (16–41).

### Arterial blood pressure

Blood pressure was measured using an automatic sphygmomanometer (HEM-7322., Omron Healthcare Co., Ltd., Kyoto, Japan) in the sitting position after 10 min of rest. Arterial blood pressure was also measured in the sitting position. All measurements were performed uniformly, on the right arm. Blood pressure was measured twice, and the average was used for data analysis.

### Blood collection

Venous blood samples were collected from all the participants between 08:00 and 09:00. Participants were required to refrain from strenuous exercise for at least one day before blood collection, and fast for at least 10 h overnight. Samples were collected in tubes that contained thrombin and heparin-neutralizing agents. After collection, the blood used for serum analysis was allowed to stand at room temperature for 30 min and centrifuged at 3500 rpm for 10 min. After centrifugation, serum samples were extracted from the respective blood collection tubes and stored at -80° C until assayed. Enzymatic methods were used to analyze triglycerides (TG), high-density lipoprotein cholesterol (HDL-C), and low-density lipoprotein cholesterol (LDL-C). Serum blood lipid levels (TG, HDL-C, LDL-C,) were analyzed by Kotobiken Medical Laboratories Inc. (Tokyo, Japan).

### Statistical analysis

All measurements were presented as mean ± standard error. The Shapiro–Wilk test was used to assess normality prior to statistical processing. A two-way repeated-measures analysis of variance (with the effects of time and group as factors) was used to compare the changes in physical activity levels between the two groups. The Bonferroni method was used for post-hoc comparisons when significant interaction effects were detected. Spearman’s correlation coefficient was calculated to determine the relationship between physical activity and blood lipid levels. An unpaired *t*-test was performed for measurements when data that were considered normal. In cases where the variance was not equal, yet normality was assumed, Welch’s *t*-test was performed. Conversely, for measurements where normality was not assumed, a Mann–Whitney test was used to compare the two groups. All statistical procedures were performed using PASW Statistics 28.0, with a significance level of < 5%.

## Results

### Participants’ characteristics

Table 1 shows the participants’ characteristics. Examination of the amount of physical activity (number of steps) showed no significant differences between the two groups regarding age, height, weight, Body Mass Index (BMI), blood pressure, or Morningness–Eveningness Questionnaire (MEQ). MVPA also showed no significant differences.

**Table 1:** Participants’ characteristics

	All (n = 51)	M step (n = 11)	A step (n = 40)	M MVPA (n = 20)	A MVPA (n = 31)
Physical characteristics					
Age (years)	71.8 ± 0.6	73.0 ± 1.0	71.5 ± 0.7	72.4 ± 0.9	71.5 ± 0.8
Height (cm)	154.7 ± 1.0	156.3 ± 2.5	154.2 ± 1.1	155.2 ± 1.7	154.3 ± 1.3
Body mass (kg)	56.1 ± 1.2	58.5 ± 3.2	55.3 ± 1.3	57.7 ± 2.0	54.9 ± 1.5
BMI (kg/m^2^)	23.5 ± 0.4	23.9 ± 1.2	23.3 ± 0.5	23.9 ± 0.7	23.2 ± 0.6
SBP (mmHg)	142.6 ± 3.1	137.9 ± 7.8	144.3 ± 3.4	137.8 ± 5.2	146.2 ± 3.9
DBP (mmHg)	80.5 ± 1.8	78.4 ± 2.5	81.2 ± 2.2	78.2 ± 2.3	82.2 ± 2.5
MEQ	55.4 ± 1.1	57.1 ± 1.4	60.1 ± 0.9	58.6 ± 1.0	60.0 ± 1.2
Physical activity					
Total (steps/day)	6273.7 ± 404.0	7382.1 ± 1046.7	5807.3 ± 392.2	6867.4 ± 767.8	5682.1 ± 389.1
Morning (steps/day)	2140.3 ± 206.6	3599.2 ± 509.4	1671.5 ± 165.9***	3108.4 ± 387.6	1286.7 ± 126.5^##^
Afternoon (steps/day)	3214.9 ± 216.2	2796.5 ± 435.3	3336.2 ± 250.8	2970.0 ± 328.0	3380.8 ± 282.5
Night (steps/day)	918.4 ± 108.9	986.4 ± 203.2	799.5 ± 78.7	1068.5 ± 233.2	816.7 ± 88.1
Rate of morning steps (%)^a^	32.8 ± 1.7	48.9 ± 2.0	28.3 ± 1.4^$$$^	42.7 ± 2.1	26.4 ± 1.5^&&&^
Rate of afternoon steps (%)^a^	52.7 ± 1.7	37.2 ± 1.7	57.0 ± 1.5^$$$^	43.7 ± 2.3	58.6 ± 1.7^&&&^
Rate of night steps (%)^a^	14.5 ± 1.1	13.9 ± 2.4	14.7 ± 1.3	13.6 ± 1.8	15.1 ± 1.4
Total MVPA (min/day)	312.9 ± 32.4	269.7 ± 83.6	307.0 ± 30.4	258.3 ± 55.2	325.2 ± 32.9
Morning MVPA (min/day)	114.3 ± 13.0	128.0 ± 40.3	104.3 ± 11.0	128.9 ± 26.9	104.4 ± 11.8
Afternoon MVPA (min/day)	133.1 ± 13.2	93.0 ± 31.3	139.7 ± 13.7*	101.1 ± 21.9	154.7 ± 15.2^##^
Night MVPA (min/day)	65.5 ± 9.0	48.7 ± 16.1	63.1 ± 7.9	64.7 ± 18.3	66.1 ± 8.7
Rate of morning MVPA (%)^a^	37.2 ± 1.5	48.7 ± 2.3	34.0 ± 1.4^$$$^	47.1 ± 1.5	30.7 ± 1.2^&&&^
Rate of afternoon MVPA (%)^a^	44.6 ± 1.4	35.0 ± 1.7	47.2 ± 1.5^$$$^	35.9 ± 1.3	50.2 ± 1.4^&&&^
Rate of night MVPA (%)^a^	18.3 ± 1.1	16.3 ± 2.8	18.8 ± 1.2	17.0 ± 2.0	19.1 ± 1.2

All data are presented as mean ± standard error, BMI: body mass index, SBP: systolic blood pressure, DBP: diastolic blood pressure, MEQ: morningness–eveningness questionnaire, MVPA: moderate-to-vigorous physical activity, M step: morning step group, A step: afternoon step group, M MVPA: morning MVPA group, A MVPA: afternoon MVPA group. ^a^The morning, afternoon, and night step rates and the morning, afternoon, and night MVPA rates show the percentage of steps and MVPA for each time period relative to the total number of steps and MVPA. ***p < 0.001, *p < 0.05 compared with the morning step counts in the A step group (Mann–Whitney). ^##^*p* < 0.01 compared with morning step counts and afternoon MVPA in the A MVPA group (Mann–Whitney). ^$$$^*p* < 0.001 vs. morning and afternoon step count rates and morning and afternoon MVPA rates in the A step group (Bonferroni post-hoc). ^&&&^*p* < 0.001 vs. morning and afternoon step count rates and morning and afternoon MVPA rates in the A MVPA group (Bonferroni for post-hoc)

### Comparison of physical activity levels

No significant differences in the total number of steps or MVPA were found between the morning and afternoon groups (Table 1). Conversely, the M step group had significantly more steps in the morning than the A step group (*p* < 0.001). The A step had significantly more MVPA in the afternoon than the M step group (*p* < 0.05, Table 1). The M MVPA group had significantly more morning steps than the A MVPA group (*p* < 0.01). The A MVPA group also had significantly more MVPA in the afternoon than the M MVPA group (*p* < 0.001, Table 1). Calculation and examination of the rate of steps and MVPA for each time period showed that the rates of morning steps and MVPA were significantly higher for the M step group than for the A step group. However, the rates of afternoon steps and MVPA were significantly lower for the M step group (*p* < 0.001) (Table 1). Additionally, the M MVPA group showed significantly higher rates of morning steps and MVPA. However, they showed significantly lower rates in afternoon steps and afternoon MVPA than the A MVPA group (*p* < 0.001) (Table 1).

### Comparison of blood lipids

**Fig. 3:**
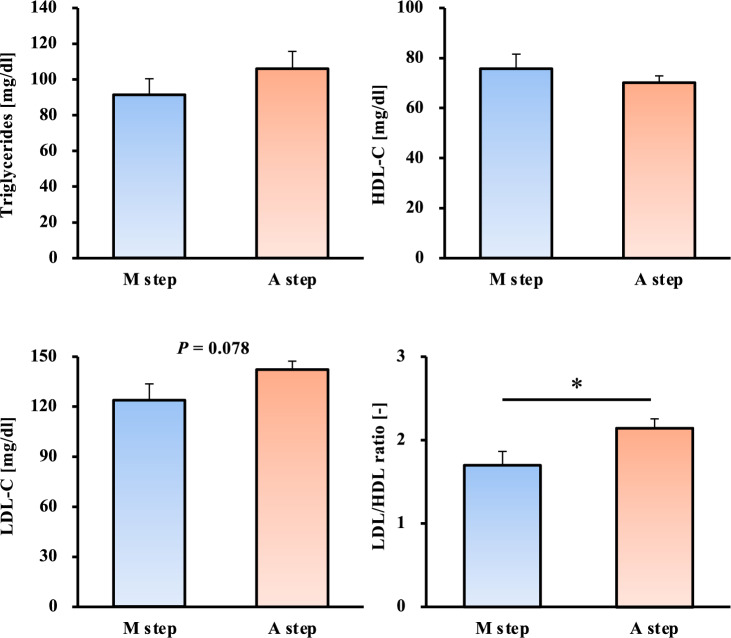
Comparison of blood lipids between the morning and afternoon step groups. HDL-C: high-density lipoprotein cholesterol, LDL-C: low-density lipoprotein cholesterol, M step: morning step group, A step: afternoon step group. All data are presented as mean ± standard error. *p < 0.05 compared to the LDL/HDL ratio in the A step group (Welch t-test)

**Fig. 4:**
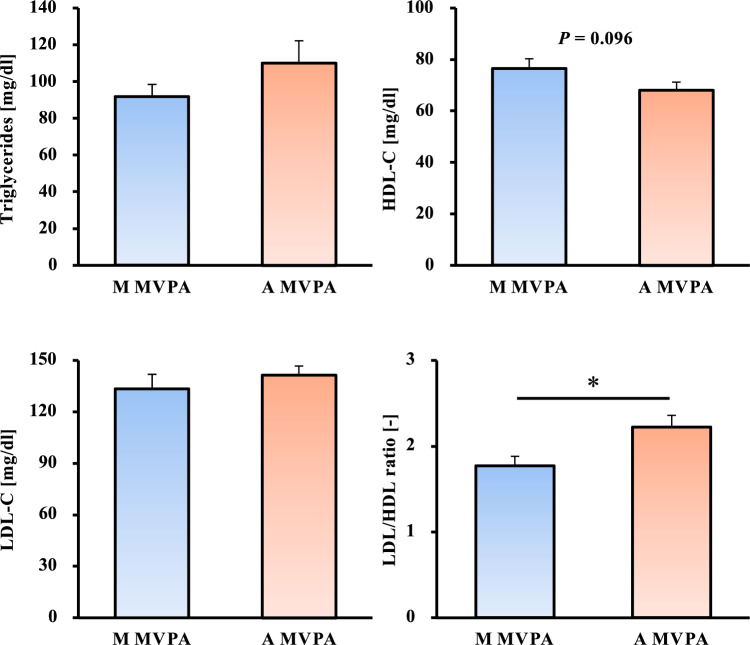
Comparison of blood lipids between the morning and afternoon MVPA groups. HDL-C: high-density lipoprotein cholesterol, LDL-C: low-density lipoprotein cholesterol, MVPA: moderate-to-vigorous physical activity, M MVPA: morning MVPA group, A MVPA: afternoon MVPA group. All data are presented as mean ± standard error. *p < 0.05 compared with the LDL/HDL ratio in the A MVPA group (*t*-test)

When examined by the number of steps, the M step group had more favorable values than the A step for all items, with a trend toward lower LDL-C values (*p* = 0.078, Fig. 3). Regarding the LDL/HDL ratio, the M step group showed significantly lower values than the A step group (*p* < 0.05, Fig. 3). Additionally, examination by MVPA showed that the M MVPA group had lower HDL-C (*p* = 0.096, Fig. 4) and significantly lower LDL/HDL ratio than the A MVPA group (*p* < 0.05, Fig. 4).

### Relationship between physical activity levels and blood lipids

No statistically significant association was found between physical activity and blood lipids. However, a positive correlation was found between total MVPA and HDL-C levels (*r* = 0.254, *p* = 0.072, Fig. S1a). A positive correlation was also observed between morning MVPA and HDL-C levels (*r* = 0.256, *p* = 0.069; Fig. S1b). Furthermore, a negative correlation was observed between morning MVPA, morning step rate, and LDL/HDL ratio (*r* = − 0.247, *p* = 0.081, *r* = − 0.248, *p* = 0.079, Fig. S1c, d).

## Discussion

To the best of our knowledge, this is the first study to evaluate the effects of objectively measured timing of the number of steps and MVPA on blood lipid outcomes in older adults. The results suggest that morning daily physical activity patterns are more beneficial for blood lipids than afternoon daily physical activity patterns.

Accumulation of morning physical activity (amount and intensity) in daily life may be effective in controlling cholesterol levels, a factor associated with vascular disease. This study showed that compared with afternoon physical activity, morning physical activity was more effective for controlling blood cholesterol levels. Many epidemiological studies and meta-analyses demonstrate the effect of physical activity on CVDs and mortality across all age groups and demographics (Kelley et al. 2005; Koba et al. 2011). Previous studies primarily focused on the total physical activity or intensity (Mann et al. 2014; Teychenne et al. 2008). Although these approaches demonstrated how certain aspects of physical activity affected the risk of CVDs, the validity of physical activity patterns under everyday conditions remains unclear. Therefore, this study examined the effects of different physical activity patterns (amount and intensity) on blood lipid levels. The M MVPA group showed a trend toward lower LDL-C and a significantly lower LDL/HDL ratio, than the A MVPA group. Furthermore, the M MVPA group had higher HDL-C levels and significantly lower LDL/HDL ratios, than the A MVPA group. Additionally, a positive correlation was observed between morning MVPA and HDL-C levels. Conversely, a negative correlation was observed between morning MVPA, morning step rate, and LDL/HDL ratio. These results suggest that the accumulation of morning physical activity in daily life play an important role in blood lipid control.

The LDL/HDL ratio is an important indicator of CVD risk. The onset and progression of CVD can be influenced by several factors. High levels of LDL-C and low levels of HDL-C are major risk factors for CVD, and have been shown to increase its incidence and mortality (Castelli et al. 1992; Downs et al. 1998; Tanne et al. 1997). LDL-C, the major transporter of cholesterol in the blood, readily permeates endothelial cell membranes when the vascular endothelium is damaged, driving plaque formation, and an inflammatory cascade that contributes to atherosclerosis (Ferrieres et al. 2018). However, HDL-C, a strong and independent predictor of CVD, strengthens the peripheral tissues of the arterial wall, prevents cholesterol deposition in the arterial wall, and promotes the recovery of damaged internal membranes (Cai et al. 2015). However, low levels of HDL-C induce inefficient elimination of cholesterol, and the development of atherosclerosis. Previous studies revealed that an elevated LDL/HDL ratio was a strong predictor of cardiovascular events in patients with coronary atherosclerosis (Amarenco et al. 2009; Barter et al. 2007; Ridker et al. 2005). Therefore, it was important to improve both HDL-C and LDL-C levels, as well as their ratio. In this study, the LDL/HDL ratio was significantly lower in the morning physical activity group (M step, M MVPA) than the afternoon physical activity group (A step, A MVPA). Previous studies reported that regular exercise in older adults reduced the risk of CVDs, such as dyslipidemia and hypertension (Chodzko-Zajko et al. 2009; Mann et al. 2014; Whelton et al. 2002). Hence, both regular exercise and the accumulation of physical activity in the morning in daily life could reduce the LDL/HDL ratio and contribute to the reduction of the risk of CVDs.

Accumulation of morning physical activity throughout the day may be important for health. In a previous study on older women, which grouped women by morning physical activity and compared obesity rates, groups with less total morning physical activity were more likely to be obese (Chomistek et al. 2016). People with chronic diseases or risk factors had shorter MVPA hours and less physical activity throughout the day. Furthermore, this tendency was more pronounced in the morning than at night (Huisingh-Scheetz et al. 2018; Sartini et al. 2015). Alterations in circadian rhythms, which regulate physiological functions, could also contribute to the risk of CVD, implying that disrupted circadian rhythms were associated with an increased incidence of CVD, diabetes, and cancer (Harfmann et al. 2015; Schroder et al. 2015). Photic (light stimuli) and non-photic (e.g., physical activity levels) environmental cues entrained or regulated circadian rhythms in humans (Mistlberger and Skene 2005; Tahara et al. 2017; Yamanaka et al. 2014). Therefore, being physically active in the morning could result in additional biologically appropriate light exposure, which could influence circadian rhythms (McFadden et al. 2014; Reid et al. 2014). In a previous study, reception of most of the average daily light exposure in the early hours was associated with lower BMI (Reid et al. 2014). Therefore, accumulating morning physical activity in daily life may contribute to the regulation of circadian factors that influence blood lipid levels, a factor associated with CVDs.

The strength of this study is the use of the accelerometer to objectively evaluate physical activity under free-living conditions. Additionally, while most studies on physical activity and exercise timing focus on and examine intensity or amount, this study explores amount and intensity simultaneously. However, this study has several limitations. The levels and patterns of physical activity vary according to sex, age, and work status (Pulakka et al. 2019; Schrack et al. 2014; Wennman et al. 2019). Physical activity among older adults was higher in women than men, and workers were more physically active than retirees (Bellettiere et al. 2015; Yerrakalva et al. 2017). As most participants in this study were older women, further detailed studies that consider these factors are required. Physical activity among older adults also decreased from morning to evening. Previous studies reported longer periods of sedentary behavior and less physical activity at night than in the morning, or during the day (Schlaff et al. 2017; Van Cauwenberg et al. 2015; Yerrakalva et al. 2017). This study also found that physical activity at night was lower than that in the morning or during the day. Therefore, a comparison with older adults who are more physically active at night was not possible. We need to increase the sample size and study this issue more closely in the future. The sample size of the current study is small. We performed a post hoc analysis and found that the power was approximately 78% with an effect size of 0.8, and an alpha of 0.05 for the sample size of 51 participants. Thus, we believe that a more detailed study with a larger sample size is needed in the future. The majority of participants in this study were older women, which may affect the interpretation of the results. Important risk factors for cardiovascular disease, such as blood pressure and blood lipids, may differ by sex and age (Maas and Appelman 2010; van den Beld et al. 2018). Therefore, more studies that take sex and age into account are needed in the future. Energy intake, which could affect blood lipid levels, was not assessed; future studies should consider the same (Gomez-Delgado et al. 2021). The accelerometer used in this study evaluates physical activity by having participants wear it for a week. However, it is still unclear whether the one-week measurements reflect long-term physical activity in daily life. Finally, the biological effects on physical activity patterns must be divided into exercise and lifestyle activities. Exercise refers to physical activities that are performed in a planned, structured, and repetitive manner to maintain or improve health. However, activities of daily living are daily dusk activities such as occupational tasks, housework, commuting to and from work, among others (Caspersen et al. 1985). Previous studies focusing on the time of day when structured exercise is performed show that compared with morning exercise, evening exercise is more effective in controlling blood glucose and blood lipids (Kim et al. 2022; Savikj et al. 2019), which differs from this study’s findings. These are attributable to diurnal variations in metabolism-related hormones controlled by the biological clock (Kim et al. 2022; Savikj et al. 2019). However, physical activity under free-living conditions also occurs during housework, commuting, and occupational tasks, and accumulates throughout the day. Therefore, the effects on the organism of the temporal distribution of physical activity accumulated throughout the day may differ from those of structured exercise. New strategies for controlling health problems associated with different physical activity patterns could be established based on the findings.

## Conclusion

In conclusion, our study indicates that the accumulation of physical activity (amount and intensity) in the morning in the daily lives of older adults is more effective than physical activity in the afternoon in lowering LDL/HDL, one of the risk factors for cardiovascular disease.

## Supplementary Information

Below is the link to the electronic supplementary material.

Supplementary file1 (PPTX 67 KB)

## Data Availability

The data supporting the results of this study are available upon request from the corresponding author, Hyeon-Ki Kim. The data are not publicly available due to restrictions that include information that could compromise the privacy of research participants.

## Abbreviations

CVDs: Cardiovascular diseases
HDL-C: High-density lipoprotein cholesterol
LDL-C: Low-density lipoprotein cholesterol
MVPA: Moderate-to-vigorous physical activity
METs: Metabolic equivalents
MEQ: Morningness–eveningness questionnaire
TG: Triglycerides
