# Supplementary material for: Morning physical activity may be more beneficial for blood lipids than afternoon physical activity in older adults: a cross-sectional study
Source: Eur J Appl Physiol. 2024 Jun 14;124(11):3253–63. doi: 10.1007/s00421-024-05526-y (PMC11519190; doi:10.1007/s00421-024-05526-y)
Supplement: Supplementary file 1 — Supplementary file1 (PPTX 67 KB) [file 421_2024_5526_MOESM1_ESM.pptx]

## Slide 1
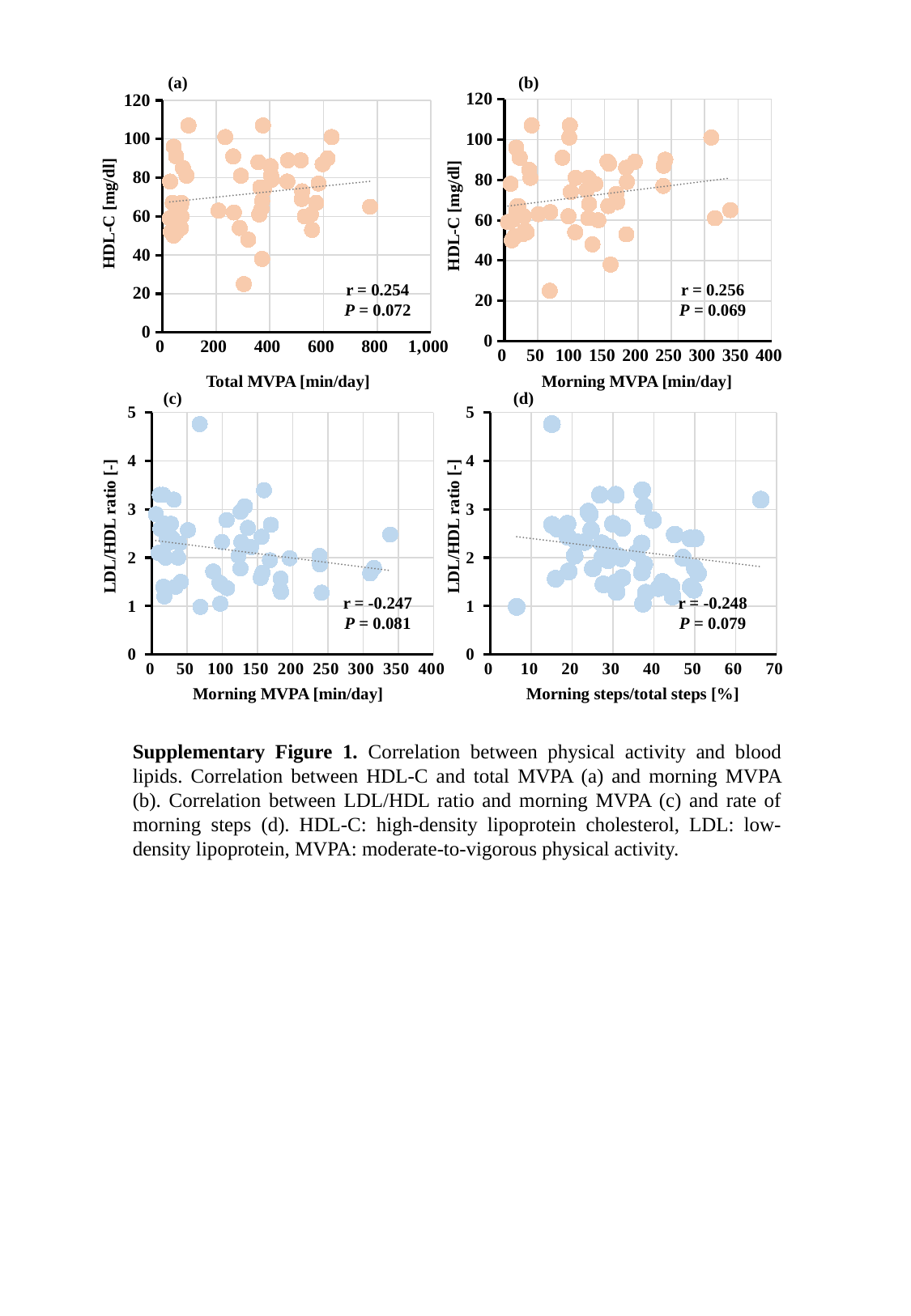

(a)
(b)
### Chart
| Category | |
|---|---|
### Chart
| Category | |
|---|---|HDL-C [mg/dl]
HDL-C [mg/dl]
r = 0.254
P = 0.072
r = 0.256
P = 0.069
Total MVPA [min/day]
Morning MVPA [min/day]
(c)
(d)
### Chart
| Category | |
|---|---|
### Chart
| Category | |
|---|---|LDL/HDL ratio [-]
LDL/HDL ratio [-]
r = -0.247
P = 0.081
r = -0.248
P = 0.079
Morning steps/total steps [%]
Morning MVPA [min/day]
Supplementary Figure 1. Correlation between physical activity and blood lipids. Correlation between HDL-C and total MVPA (a) and morning MVPA (b). Correlation between LDL/HDL ratio and morning MVPA (c) and rate of morning steps (d). HDL-C: high-density lipoprotein cholesterol, LDL: low-density lipoprotein, MVPA: moderate-to-vigorous physical activity.
